# Esophageal Cancer in Young People: A Case Series of 109 Cases and Review of the Literature

**DOI:** 10.1371/journal.pone.0014080

**Published:** 2010-11-22

**Authors:** Sonja P. Dawsey, Stanley Tonui, Robert K. Parker, John W. Fitzwater, Sanford M. Dawsey, Russell E. White, Christian C. Abnet

**Affiliations:** 1 Nutritional Epidemiology Branch, Division of Cancer Epidemiology & Genetics, National Cancer Institute, Bethesda, Maryland, United States of America; 2 Tenwek Hospital, Bomet, Kenya; 3 Department of Surgery, Rhode Island Hospital and The Warren Alpert Medical School of Brown University, Providence, Rhode Island, United States of America; 4 Department of Surgery, Texas Tech University School of Medicine, Health Sciences Center, Lubbock, Texas, United States of America; The University of Hong Kong, Hong Kong

## Abstract

Certain geographically distinct areas of the world have very high rates of esophageal cancer (EC). Previous studies have identified western Kenya as a high risk area for EC with an unusual percentage of cases in subjects 30 years of age or younger. To better understand EC in these young patients, we abstracted available data on all 109 young patients diagnosed with EC at Tenwek Hospital, Bomet District, Kenya from January 1996 through June 2009, including age at diagnosis, sex, ethnicity, tumor histology, residence location, and medical interventions. We also attempted to contact all patients or a family member and obtained information on ethnicity, tobacco and alcohol use, family history of cancer, and survival. Sixty (55%) representatives of the 109 young patients were successfully interviewed. The median survival time of these 60 patients was 6.4 months, the most common tumor histology was esophageal squamous cell carcinoma (ESCC) (98%), the M:F ratio was 1.4∶1, and only a few subjects used tobacco (15%) or alcohol (15%). Seventy-nine percent reported a family history of cancer and 43% reported having a family history of EC. In summary, this case series describes the largest number of young EC patients reported to date, and it highlights the uniqueness of the EC experience in western Kenya.

## Introduction

Worldwide, esophageal cancer (EC) ranks eighth in cancer incidence and sixth in cancer mortality [1]. There are two primary cell types of EC, esophageal adenocarcinoma (EAC) and esophageal squamous cell carcinoma (ESCC); together these two types account for >95% of all cases of EC. In recent years, EAC rates have increased in most Western industrialized countries, and it has become the predominant form of EC in these populations; however, in other areas of the world, ESCC still predominates. About 80% of ECs occur in developing countries, and in these countries, nearly all of these cancers are ESCC [1].

The incidence of EC varies widely, and certain areas such as northern China [1], northeastern Iran [2], and South Africa [3] have very high rates of this disease, with age-standardized incidence rates from 50 to over 100 cases per 100,000 population per year. In contrast, most Western countries have much lower incidence rates of EC, from 4 to 10 cases per 100,000 population per year [4], [5].

Western Kenya also appears to have high rates of esophageal cancer. It has proven difficult to establish reliable cancer or death registries in this area, but case series reports from Tenwek Hospital, a tertiary care center in southwestern Rift Valley Province, and Moi Teaching and Referral Hospital, a tertiary care center in northern Rift Valley Province, show that EC is the most common cancer [6], [7], [8].

In both low- and high-incidence areas, EC is rare in individuals younger than age 30. In the US, the mean age of EC patients at diagnosis is 68 [4], and it rarely presents ≤30 years of age. EC cases in those ≤30 years of age in northern China, northeastern Iran, and the SEER registries in the US account for 0.7%, 1%, and 05% of cases, respectively [9], [10], [4]). At Tenwek Hospital, however, 6.3% of all EC cases are ≤30 [7]. To better understand the unusually frequent occurrence of EC in young people in this area, we conducted a retrospective study of all of the young EC patients diagnosed at Tenwek Hospital between January 1996 and June 2009.

## Methods

### Subject Identification and Data Gathering

We examined all pathology reports, endoscopy records, and patient files from Tenwek Hospital from January 1996 through June 2009 to identify all patients with a histologic or endoscopic diagnosis of EC who were ≤30 years of age (considered “young EC patients”). During this 13.5 year period, 109 such young EC patients were identified. We reviewed the following from the records of these patients: age at diagnosis, sex, ethnicity (specifically, tribal background), tumor histology, last known residence, and treatments.

To supplement the chart review and better understand the clinical course of EC in young patients at Tenwek, we attempted to locate all patients, their living family members, or another proxy familiar with their medical history. We successfully located a respondent for 60 of the 109 patients. Respondents were interviewed in their homes by a trained interviewer, using a structured questionnaire to obtain information on demographic characteristics, lifestyle, family history, and survival.

This study was approved by the human subjects review committee of Tenwek Hospital, and analysis of anonymized data was exempted from review by the Office of Human Subjects Research at the US National Cancer Institute.

### Statistical analysis

The residence location of each subject was determined using the global positioning system coordinates from the GEOnet Names Server (http://www.nga.mil) and was mapped using Epi Info version 3.4.3 (CDC) software. Kaplan-Meir curves and median survival times were estimated using SAS 9.1 (SAS Institute, Inc, Cary, NC). Follow-up time was calculated using date of initial diagnosis and date of death. Date of initial diagnosis was identified from medical records. Date of death was obtained from medical records or interview responses.

### Literature review

The literature was abstracted using the MEDLINE and PubMed databases (National Library of Medicine), initially using keywords: “esophageal cancer young” and/or “esophageal carcinoma young” with limits of: Humans, Case Reports, Core clinical journals, Cancer, MEDLINE, PubMed Central, All Infant: birth-23 months, All Child: 0–18 years, All Adult: 19+ years, Newborn: birth-1 month, Infant: 1–23 months, Preschool Child: 2–5 years, Child: 6–12 years, Adolescent: 13–18 years. Other keywords included combinations with: childhood cancer of the esophagus, young squamous cell carcinoma, young adenocarinoma, barrett's esophagus, and adolescence. Reference lists of all selected references were used as a secondary source. The search yielded 37 useful articles with 145 reports of esophageal malignancies in patients ≤30 years of age.

## Results

**Figure 1:**
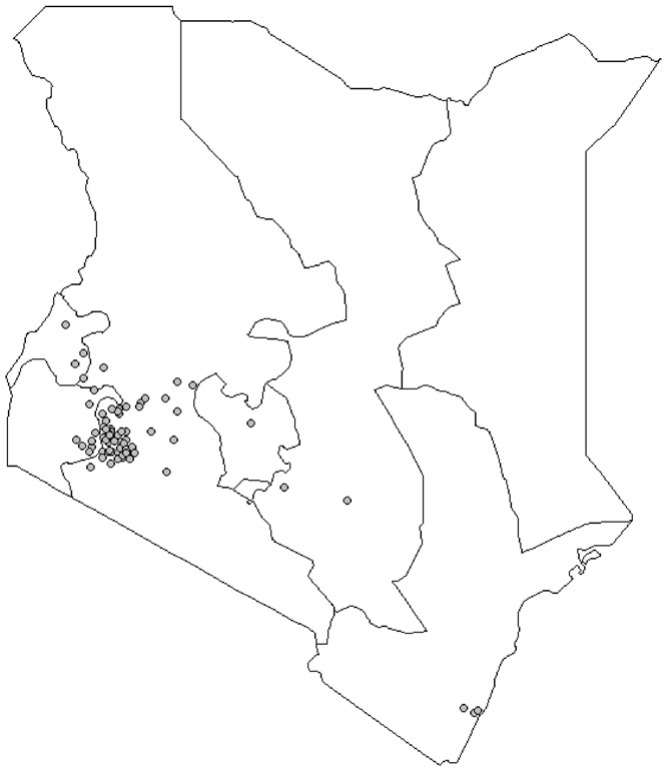
Home villages of young esophageal cancer patients. Locations of the home villages of the 109 esophageal cancer patients ≤30 years of age seen at Tenwek Hospital (star) from January 1996 through June 2009.

Between 1996 and 2009, 109 patients 30 years of age or younger were diagnosed with EC at Tenwek Hospital, with the youngest subject 14 years of age. This included 65 males and 44 females, a M:F ratio of 1.5∶1 (**Table 1**). Eighty-seven (95%) of the 92 cases with known histology were ESCCs. Eighty percent of the young patients were of the Kalenjin ethnic group. **Figure 1** is a map showing the residence locations of all of the 109 patients.

**Table 1:** Distributions of 109 esophageal cancer patients ≤30 years of age seen at Tenwek Hospital from January 1996 through June 2009.

N	109
Age, years, mean (SD)	25 (4)
Sex, M:F	1.5∶1
Male, N (%)	65 (60)
Female, N (%)	44 (40)
Histology	
Known, N (%)	92 (84)
ESCC, N (%)	87 (95)
EAC, N (%)	5 (5)
Unknown, N (%)	17 (16)
Ethnic group	
Kalenjin, N (%)	87 (80)
Non-Kalenjin, N (%)	22 (20)

**Figure 2:**
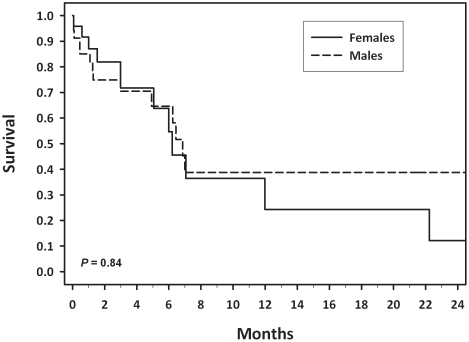
Survival with esophageal cancer in young patients by sex. Survival by sex of the 60 esophageal cancer patients ≤30 years of age seen at Tenwek Hospital from January 1996 through June 2009 who had follow-up information.

We successfully collected follow-up information on 60 (55%) of the 109 young patients. In the subgroup with follow-up information (**Table 2**), the M:F ratio was 1.4∶1, 98% were pathology-confirmed ESCC cases, and 95% were part of the Kalenjin ethnic group, which is composed of seven related tribes living in southwestern Kenya. Thirty-five (58%) of the 60 patients elected to forgo palliative therapy (**Table 2**). Twenty-one subjects (35%) received palliative interventions. Only four cases (7%) were candidates for esophagectomy, and one survived more than 5 years. Most patients had short survival times, and there was no significant difference by sex (*P* = 0.84), with median survival times of 6.9 months in males and 6.2 months in females (**Figure 2**).

**Table 2:** Distributions of 60 esophageal cancer patients ≤30 years of age seen at Tenwek Hospital from January 1996 through June 2009 who had follow-up information.

N	60
Age, years, mean (SD)	25 (4)
Sex, M:F	1.4∶1
Male, N (%)	35 (58)
Female, N (%)	25 (42)
Histology	
ESCC, N (%)	59 (98)
EAC, N (%)	1 (2)
Ethnic group	
Kalenjin, N (%)	57 (95)
Non-Kalenjin, N (%)	3 (5)
Interventions	
Stent, N (%)	14 (23)
Esophagectomy, N (%)	4 (7)
Other, N (%)	7 (12)
None, N (%)	35 (58)
Survival	
Range, days	2 – 2920
Median, months	6.4
Alive at end of follow-up (%)	3 (5)
Survival unknown, N (%)	8 (13)

We examined several known risk factors for EC in the 60 followed patients (**Table 3**). None of the followed female patients (n = 25) smoked tobacco, and only one had ever consumed alcoholic beverages. Among the male patients (n = 35), 9 (26%) smoked tobacco and 8 (23%) drank alcoholic beverages. A family history of cancer was present in 45 (79%) of the 57 subjects in which such a history was known, and there was a family history of EC in 21 (43%) of the 49 subjects with such data, including 5 (10%) with multiple EC cases in their families.

**Table 3:** Distributions of risk factors overall and by sex for esophageal cancer among patients ≤30 years of age seen at Tenwek Hospital from January 1996 through June 2009 who had follow-up information.

	Total	Male	Female
N	60	35	25
Tobacco smoking			
Yes, N (%)	9 (15)	9 (26)	0 (0)
No, N (%)	51 (85)	26 (74)	25 (100)
Alcoholic beverage drinking			
Yes, N (%)	9 (15)	8 (23)	1 (4)
No, N (%)	51 (85)	27 (77)	24 (96)
Family history of cancer			
Known, N (%)	57 (95)	32 (91)	25 (100)
Yes, N (%)	45 (79)	23 (72)	22 (88)
No, N (%)	12 (21)	9 (28)	3 (12)
In first degree relative, N (%)	25 (44)	10 (31)	15 (60)
Multiple CA family hx, N (%)	16 (28)	6 (19)	10 (40)
Unknown, N (%)	3 (5)	3 (9)	0 (0)
Family history of esophageal cancer			
Known, N (%)	49 (82)	28 (80)	21 (84)
Yes, N (%)	21 (43)	9 (32)	12 (57)
No, N (%)	28 (57)	19 (68)	9 (43)
Multiple EC family hx, N (%)	5 (10)	2 (7)	3 (14)
Unknown, N (%)	11 (18)	7 (20)	4 (16)

A search of the literature found 37 articles describing 145 cases of EC patients 30 years of age or younger. 102 (70%) of the cases were in case series in which the exact ages were not indicated, and for 104 cases (72%), the tumor histology was not given (**Table 4**). Of the 43 cases with reported ages, the median age was 17 years and the age range was from 8 years to 30 years. The M:F ratio in the 145 reported cases was 1.8∶1. Of the 41 cases with reported histology, 17 (41%) were ESCC, 16 (39%) were EAC, and 8 (20%) reported as EC not otherwise specified (NOS) (**Table 5**).

**Table 4:** Published papers presenting information on esophageal cancer in young patients.

					Histology			
Date	Reference	No. Cases	Mean Age	No. Males	No. ESCC	No. EAC	No. EC NOS	Location
1925	Jackson [24]	2	23		unknown			USA
1929	Kaufuman [25]	1	21	0	0	0	1	Germany
1955	Saettler [26]	1	24	0	0	0	1	Germany
1961	Hahlbrock [27]	1	13	1	0	0	1	Germany
1963	Birzel [28]	1	12	1	0	0	1	Germany
1967	Sanowaski [29]	1	24	1	1	0	0	USA
1967	Wright [30]	2	21	2	1	0	1	England
1968	Kinnman [31]	1	15	1	1	0	0	Korea
1968	Paymaster * [23]	86	25	58	unknown			India
1971	Das * [32]	11	≤30	3	unknown			India
1976	Oberiter [33]	1	12	0	0	0	1	Croatia
1977	Morota [34]	1	18	1	0	0	1	Japan
1977	Poleynard [35]	1	25	1	0	1	0	USA
1979	Tata [36]	1	17	0	0	0	1	India
1979	Singh [37]	1	14	1	1	0	0	India
1980	Soni [38]	1	8	0	1	0	0	India
1983	Elliott [39]	1	14	1	0	1	0	England
1984	Hilou [40]	1	15	1	0	1	0	England
1986	Bright [41]	1	20	1	0	1	0	Australia
1988	Khastgir [42]	1	18	0	1	0	0	India
1988	Dewar [43]	1	20	0	1	0	0	Australia
1989	Adzick [44]	1	20	0	0	1	0	USA
1989	Shahi [45]	1	14	1	1	0	0	India
1989	Hoeffel [46]	2	13	2	0	2	0	France
1992	Kumar [47]	7	17	5	4	3	0	India
1993	Hassall [48]	1	17	1	0	1	0	Canada
1993	Aryya [49]	1	10	1	1	0	0	India
1997	Gangopadhyay [50]	1	8	1	0	1	0	India
1998	Schettini [51]	1	11	0	1	0	0	Brazil
1999	Karwasra [52]	1	17	1	1	0	0	India
2001	Singh [53]	1	18	1	1	0	0	India
2001	Zotter [54]	1	16	1	0	1	0	Austria
2003	Al-Hilli * [55]	5	25	3	unknown			Bahrain
2005	Pultrum [56]	1	22	0	0	1	0	Netherlands
2005	Tampi [57]	1	15	1	1	0	0	India
2007	Moreels [58]	1	28	1	0	1	0	Netherlands
2007	Shinohara [59]	1	27	0	0	1	0	USA

*These references did not give exact ages, so the center of the range is given.

**Table 5:** Summary of age, sex and histologic data from published reports of esophageal cancer in young persons, overall and separately in developing and developed countries.

	Total	Developing countries	Developed countries
Cases, N (%)	145	122 (84)	23 (16)
Sex			
Male, N (%)	92 (64)	77 (63)	15 (71)
Female, N (%)	51 (46)	45 (37)	6 (29)
M:F	1.8∶1	1.7∶1	2.5∶1
Histology			
Known, N (%)	41 (28)	20 (16)	21 (91)
ESCC cases, N (%)	17 (41)	14 (70)	3 (14)
ACA cases, N (%)	16 (39)	4 (20)	12 (57)
EC NOS, N (%)	8 (20)	2 (10)	6 (29)
Unknown, N (%)	104 (72)	102 (84)	2 (9)

One hundred twenty-two (84%) of the young EC cases reported in the literature lived in developing countries, including 114 cases (79%) in India alone. The M:F ratio was 1.7∶1 in developing countries and 2.5∶1 in developed countries. Of the cases with a specified histological cell type, ESCC predominated in the developing countries (14/18, 78%), whereas EAC predominated in developed countries (12/15, 80%) (**Table 5**).

## Discussion

Western Kenya has been identified as an area with a common occurrence of ESCC. Of patients that are diagnosed at Tenwek Hospital, about 6% are ≤30 years of age [7]. This high percentage has not been reported anywhere else in the world. From January 1996 – June 2009, 109 such young EC cases were identified in this case series from Tenwek Hospital.

Among the total 109 cases and the 60 cases with follow-up information, the M:F ratio was close to 1.5∶1. This is similar to the gender distribution of cases found in all EC patients seen at Tenwek (1.6∶1) [7] and in other high-risk populations in developing areas, such as Linxian, China [11] and Golestan Province in northwestern Iran, [12]. This M:F ratio is much lower than those found in industrialized countries [13]. By far the most common histologically confirmed tumor type among the young EC patients seen at Tenwek was ESCC (95%), which is also the most common tumor type in adult patients at Tenwek [7] and in other known high risk areas [14]. This large percentage of ESCC may even be an underestimate, because some of the small number of EAC cases identified at Tenwek Hospital may have originated in the gastric cardia.

Two primary risk factors for esophageal cancer in Western populations are smoking tobacco and drinking alcoholic beverages in excess [15]. We found that tobacco and alcohol consumption were reported by only a minority of young EC cases at Tenwek, which supports the argument that although these exposures are associated with EC in developed countries, they do not seem to be major etiologic factors in this area. This finding is similar to other developing, high-risk ESCC areas in China [16] and Iran [17]. Notably, almost 80% of patients in this case series had a family history of cancer, including a 43% with a specific family history of EC, which is a higher percentage than in cases from a high risk area in China [16] but lower than in cases from a high-risk area in Iran [18]. The contribution of other risk factors will require formal etiologic studies, but may include consumption of very hot tea [19], limited diet [20], exposure to polycyclic aromatic hydrocarbons [21], or genetics.

It is also important to note that most young patients in this series were of Kalenjin ethnicity, although the meaning of this is difficult to assess in a case-series. A similar proportion of Kalenjins has been reported among EC patients from the traditional catchment area around Tenwek Hospital [7]. The high proportion of cases with a family history of EC and the apparent restriction to a specific ethnic background both suggest that genetic factors could be important in the etiology of EC in this area, but these observations could also reflect shared environmental risk factors such as socioeconomic status, diet, use of similar traditional medicines [22] or foods, or communicable diseases.

Of the followed patients, survival was poor, with a median of 6.4 months, which is shorter than the still poor survival of 9.2 months seen for all EC cases in the United States [13]. In all populations, the majority of EC cases are diagnosed at an advanced stage, and it appears that this is especially true among young patients at Tenwek. Local knowledge of the high case fatality rate may further discourage cases from coming to the hospital until the cancer is very advanced.

Our literature review shows that little is known about EC in young people in any population. We found several case series of young patients from India, but only limited reports from other countries. Taken together, these reports suggest that the occurrence of EC in patients ≤30 years of age is rare throughout the world; even in the large case series presented by Paymaster *et al* [23] young EC patients comprised only around 1% of their cases. These literature reports also suggest that the demographic and tumor characteristics of EC in young patients are similar to those of EC in older patients from the same populations: the M:F ratio in the reported young patients was close to one (1.8∶1) in cases from developing countries and was greater (2.5∶1) in cases from developed countries, and the proportion of ESCC tumors was high (14/18, 78%) in cases from developing countries and it was low (3/15, 20%) in cases from developed countries.

In summary, this case series describes the largest number of young EC patients reported to date, and it highlights the uniqueness of the EC experience in western Kenya. The causes of the overall high incidence and the particularly high incidence in young people remain unknown and will require detailed epidemiologic studies of the local population.
